# Swept Mechanism of Micro-Milling Tool Geometry Effect on Machined Oxygen Free High Conductivity Copper (OFHC) Surface Roughness

**DOI:** 10.3390/ma10020120

**Published:** 2017-01-28

**Authors:** Zhenyu Shi, Zhanqiang Liu, Yuchao Li, Yang Qiao

**Affiliations:** 1 Key Laboratory of High Efficiency and Clean Mechanical Manufacture, Shandong University, Ministry of Education, Jinan 250061, China; shizhenyu@sdu.edu.cn (Z.S.); sduliyuchao2014@gmail.com (Y.L.); 2 School of Mechanical Engineering, Shandong University, Jinan 250061, China; 3 School of Mechanical Engineering, University of Jinan, Jinan 250061, China; me_qiaoy@ujn.edu.cn

**Keywords:** surface roughness, sweeping volume, tool geometry, micro-end milling

## Abstract

Cutting tool geometry should be very much considered in micro-cutting because it has a significant effect on the topography and accuracy of the machined surface, particularly considering the uncut chip thickness is comparable to the cutting edge radius. The objective of this paper was to clarify the influence of the mechanism of the cutting tool geometry on the surface topography in the micro-milling process. Four different cutting tools including two two-fluted end milling tools with different helix angles of 15° and 30° cutting tools, as well as two three-fluted end milling tools with different helix angles of 15° and 30° were investigated by combining theoretical modeling analysis with experimental research. The tool geometry was mathematically modeled through coordinate translation and transformation to make all three cutting edges at the cutting tool tip into the same coordinate system. Swept mechanisms, minimum uncut chip thickness, and cutting tool run-out were considered on modeling surface roughness parameters (the height of surface roughness *R_z_* and average surface roughness *R_a_*) based on the established mathematical model. A set of cutting experiments was carried out using four different shaped cutting tools. It was found that the sweeping volume of the cutting tool increases with the decrease of both the cutting tool helix angle and the flute number. Great coarse machined surface roughness and more non-uniform surface topography are generated when the sweeping volume increases. The outcome of this research should bring about new methodologies for micro-end milling tool design and manufacturing. The machined surface roughness can be improved by appropriately selecting the tool geometrical parameters.

## 1. Introduction 

The miniaturization of devices demands the production of mechanical components with manufactured features in the range of a few to a few hundred microns [1]. As devices are being more miniaturized, the ratio of surface area to volume (i.e., the size of devices) increases, resulting in surface effects playing a more important role in the performance [2]. Surface roughness is predominantly considered as the most important feature of practical engineering surfaces due to its crucial influence on the mechanical and physical properties of the machined parts [3,4]. Therefore, it is particularly necessary to understand the effects of manufacturing process parameters on the surface characteristics when miniaturized components are fabricated.

Micro-milling is among the principal manufacturing processes which allows the development of components possessing micrometric dimensions, being used for the manufacture of both forming tools and the final product [5,6]. A number of studies have been reported concerning the effect of cutting parameters on surface topography through experimental efforts. Weule et al. [7] studied the machined surface generated in the micro-cutting of steel. They found that the surface roughness increased at a lower feed rate relative to the cutting edge radius. In addition, the surface quality of the steel workpiece was strongly influenced by the workpiece microstructure and the cutting velocity. In their study, rougher surfaces were produced by tools with larger edge radii. Yuan et al. [8] also studied the effect of the cutting edge radius on the surface roughness of aluminum alloys with diamond tools in turning operations. The cutting edge radii of the cutting tools used in this research were 0.3 μm and 0.6 μm. The results showed that the diamond tool sharpness had a considerable influence on the machined surface integrity. When the cutting speed and feed rate used were 314 m/min and 2.5 μm/rev, results showed that the sharper the cutting edge was (that is the smaller cutting edge radius), the better a smaller surface roughness can be got. Son et al. [9] conducted experiments to investigate the characteristics of micro cutting under various depth-of-cuts from 0.1 to 0.5 µm and a fixed feed speed of 3 mm/s. Results showed that, the smallest surface roughness for Oxygen Free High Conductivity Copper (OFHC) was obtained at 0.1 µm depth of cut due to the generation of continuous chip. While for aluminum machining, continuous chip and the smallest roughness were produced at a minimum cutting thickness of 0.2 µm. The smallest surface roughness for OFHC was generated at the 0.1 µm depth of cut, but it was supposed that this surface was produced not by cutting but by burnishing. Therefore, it was concluded that a continuous chip was generated and the surface quality was the best when cutting at the minimum cutting thickness.

Several researchers studied various theoretical bases for analytical models to investigate the effects of cutting parameters on surface topography. Burlacu and Iordan [10] developed a mathematical model to investigate the effects of cutting tool diameter, cutting speed, depth-of-cut, and feed rate on surface roughness by using a multiple regression method. Kiswanto et al. [11] researched the effect of spindle speed, feed rate, and machining time on the surface roughness and burr formation of aluminum alloy during the micro milling operation. Campatelli [12] adopted the response surface method to optimize the process parameters for minimizing power consumption in the milling of carbon steel. Kant and Sangwan [13] used an artificial neural network coupled with a Genetic Algorithm for predictive modelling and optimization of machining parameters to minimize surface roughness.

Most of the previous studies on surface integrity [14,15,16,17,18,19,20] have assumed that the geometric surface finish was influenced by the cutting speed, feed rate, and cutting edge radius. In addition, the surface roughness is also affected by depth-of-cut, tool wear, tool natural frequency, presence of BUE (built-up-edge), workpiece hardness [21,22,23,24,25], etc. The whole nose of the cutting tool actually participates in the micro-cutting process because the metal removal volume is very small. The influence of the mechanism of cutting tool geometry on the surface roughness in micro-cutting when both the cutting tool edge radius and the tool nose are involved in the cutting process is still not clear.

This paper aims to investigate the influence of the mechanism of cutting tool parameters on surface topography based on the analysis of a number of surfaces fabricated through the micro milling process. The cutting tool parameters include the cutting edge radius, the flute number, and the helix angle. A comprehensive theory is established to explain the quantitative relationship between parameters and surface topography. The established methodology model can clarify the importance of cutting tool parameters on preparation of a desired surface.

**Figure 1:**
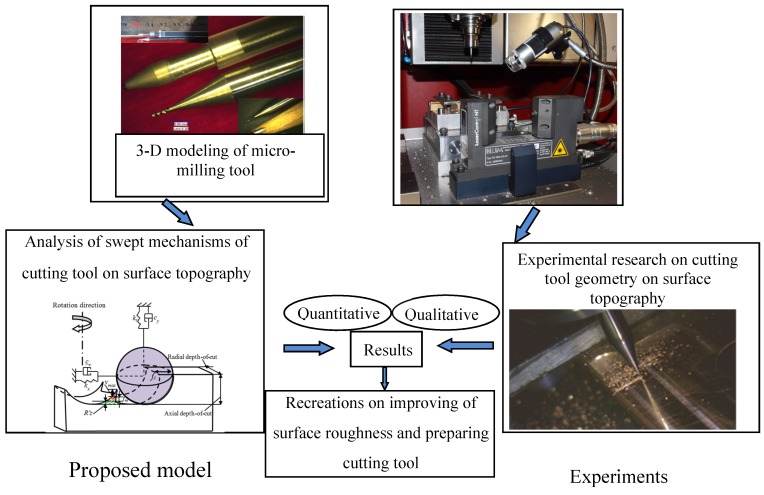
Scope of this research.

In this paper, four different cutting tool geometries including two flutes with helix angle 15° and 30°, respectively, three flutes with helix angle 15° and 30°, respectively, are examined to determine the effects of cutting tool geometry on the surface topography by combining theoretical and experimental analyses. The general framework of this research is shown in Figure 1.

## 2. Swept Mechanisms of Tool Geometry Effect on Surface Topography

### 2.1. Three Dimensional Geometrical Modeling of Micro-Milling Tool

**Figure 2:**
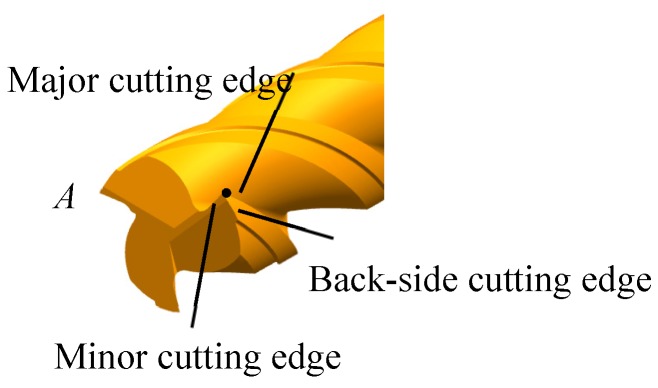
General view of cutting edge of milling cutter.

In the micro-milling process, the actual geometry involved in the process is determined by the major cutting edge, the minor cutting edge, and the third edge called the back-side cutting edge as shown in Figure 2 [26]. All of these three cutting edges should be considered when modeling the actual geometry of the cutting tool involved in the micro-machining.

The mathematic models of the actual geometry of the cutting tool involved in machining can be developed through coordinate translation and transformation [27].

**Figure 3:**
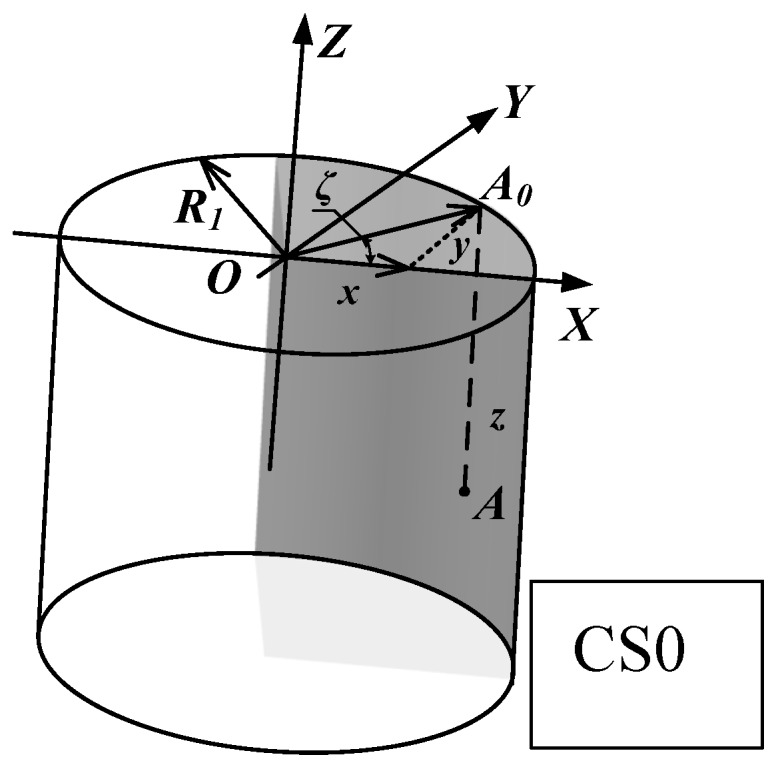
Minor cutting edge in coordinate system *O*-*XYZ*.

Three different coordinate systems are used for modeling the description. The *CS*0 is a global coordinate system of the cutting tool. Point *A* existing in the cutting tool tip as shown in Figure 2 is represented on the coordinate system *O*-*XYZ* as shown in Figure 3. The coordinate system is established with the coordinate origin *O* at the center of the circular-arc of the minor-cutting edge [28]. The *Z*-axis is set to be the direction of the minor cutting edge. The *X*-axis is set in a direction perpendicular to the tangent line of the minor cutting edge. The *Y*-axis is set perpendicular to the *X*- and *Z*-axes.

Then the point *A* at the minor-cutting edge can be expressed in Equation (1).

(1) $$A\left(\zeta ,k\right)=\{\begin{array}{c} x=R_{1}\cos\zeta \\ y=R_{1}\sin\zeta \\ z=-k \end{array}$$

where 0 < *k* < *l*; *l* is the minor cutting edge length; *R*_1_ is the minor cutting edge radius; ζ is the angle between *OA*_0_ and *OX*; and *A*_0_ is the projection of point *A* in the *XOY* plane.

**Figure 4:**
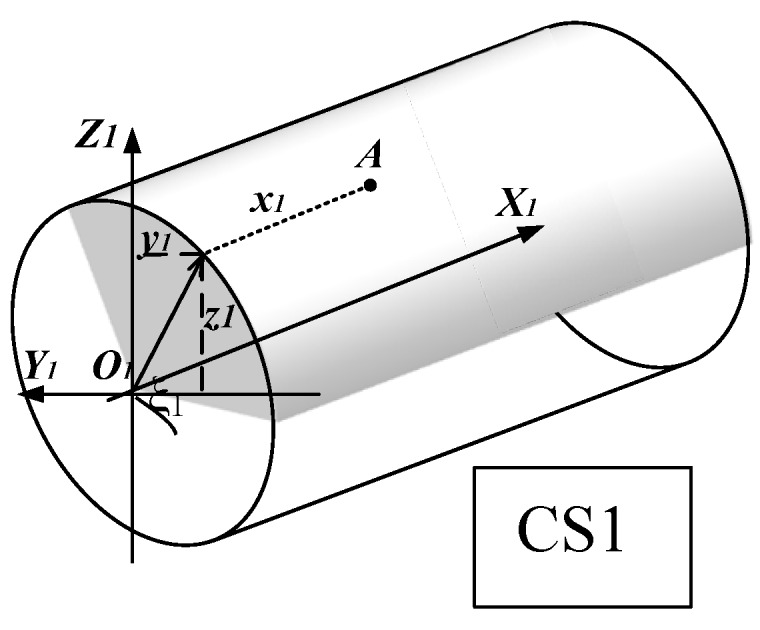
Major cutting edge in coordinate system *O*_1_-*X*_1_*Y*_1_*Z*_1_.

Figure 4 shows point A on the major cutting edge in the coordinate system *O*_1_-*X*_1_*Y*_1_*Z*_1_, where *CS*1 are the local coordinates. The coordinate origin *O*_1_ is at the center of the circular-arc of the major-cutting edge.

The major cutting edge in coordinate system *O*_1_-*X*_1_*Y*_1_*Z*_1_ can be expressed in Equation (2).

(2) $$A\left(\zeta_{1}，u_{1}\right)=\left[\begin{array}{c} x_{1}\\ y_{1}\\ z_{1} \end{array}\right]=\left[\begin{array}{c} u_{1}\\ -r_{1}\cos\zeta_{1}\\ r_{1}\sin\zeta_{1} \end{array}\right]$$

where, 0 < *u*_1_ < *m*. *m* is the major cutting edge length. *r*_1_ is the major cutting edge radius. ζ_1_ is the angle between *Y*_1_*O*_1_ and *O*_1_*A*_1_. *A*_1_ is the projection of the point *A* in the *X*_1_*O*_1_*Y*_1_ plane.

The helix angle of the milling cutting tool is γ, the coordinate system *O*_1_-*X*_1_*Y*_1_*Z*_1_ transformed to the coordinate system *O*-*XYZ* can be realized by turning around the *Z* axis for *γ* angle. The transformation matrix can be expressed by Equation (3).

(3) $$M_{1}=\left[\begin{array}{ccc} \cos\gamma & -\sin\gamma & 0\\ \sin\gamma & \cos\gamma & 0\\ 0 & 0 & 1 \end{array}\right]$$

Hence the major cutting edge in the coordinate system *O-XYZ* can be expressed by Equation (4).

(4) $$\left[\begin{array}{c} x\\ y\\ z \end{array}\right]=\left[\begin{array}{ccc} \cos\gamma & -\sin\gamma & 0\\ \sin\gamma & \cos\gamma & 0\\ 0 & 0 & 1 \end{array}\right]\cdot \left[\begin{array}{c} u_{1}\\ -r_{1}\cos\zeta_{1}\\ r_{1}\sin\zeta_{1} \end{array}\right]=\left[\begin{array}{c} u_{1}\cos\gamma +r_{1}\cos\zeta_{1}\sin\gamma \\ u_{1}\sin\gamma -r_{1}\cos\zeta_{1}\cos\gamma \\ r_{1}\sin\zeta_{1} \end{array}\right]$$

**Figure 5:**
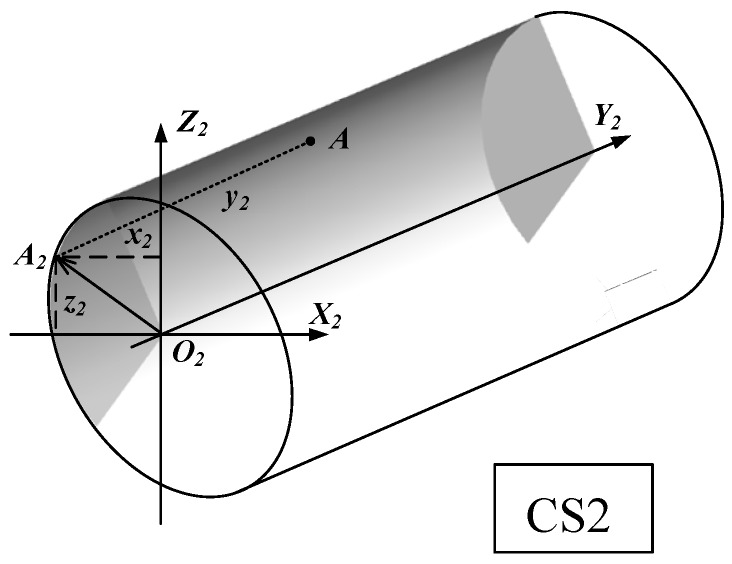
Back-side cutting edge in coordinate system *O*_2_-*X*_2_*Y*_2_*Z*_2_.

Figure 5 shows the point A at the back-side of the cutting edge in the coordinate system *O*_2_-*X*_2_*Y*_2_*Z*_2_, where *CS*2 are other local coordinates.

According to Figure 5, the point A at the back-side of the cutting edge in the coordinate system *O*_2_-*X*_2_*Y*_2_*Z*_2_ can be expressed in Equation (5).

(5) $$A\left(\zeta_{2}，u_{2}\right)=\left[\begin{array}{c} x_{2}\\ y_{2}\\ z_{2} \end{array}\right]=\left[\begin{array}{c} -r_{2}\cos\zeta_{2}\\ u_{2}\\ r_{2}\sin\zeta_{2} \end{array}\right]$$

where 0 < *u*_2_ < *n*. *n* is the length of the back-side cutting edge. *r*_2_ is the back-side cutting edge radius. ζ_2_ is the angle between *O*_2_*X*_2_ and *O*_2_*A*_2_. *A*_2_ is the projection of point *A* in the *X*_2_*O*_2_*Y*_2_ plane.

For milling tools with different flutes, the cutting edge angle between the minor-cutting edge and the back-side cutting edge will be different. In this paper, the cutting edge angle is set to be *δ*. The coordinate system *O*_2_-*X*_2_*Y*_2_*Z*_2_ is transformed by turning *O*-*XYZ* around the *Z* axis for *δ* degrees. The transformation matrix could be expressed by Equation (6).

(6) $$M_{2}=\left[\begin{array}{ccc} \cos\delta & -\sin\delta & 0\\ \sin\delta & \cos\delta & 0\\ 0 & 0 & 1 \end{array}\right]$$

The back-side cutting edge in the coordinate system *O*-*XYZ* is expressed in Equation (7).

(7) $$\left[\begin{array}{c} x\\ y\\ z \end{array}\right]=\left[\begin{array}{ccc} \cos\delta & -\sin\delta & 0\\ \sin\delta & \cos\delta & 0\\ 0 & 0 & 1 \end{array}\right]\cdot \left[\begin{array}{c} -r_{2}\cos\zeta_{2}\\ u_{2}\\ r_{2}\sin\zeta_{2} \end{array}\right]=\left[\begin{array}{c} -r_{2}\cos\zeta_{2}\cos\delta -u_{2}\sin\delta \\ -r_{2}\cos\zeta_{2}\sin\delta +u_{2}\cos\delta \\ r_{2}\sin\zeta_{2} \end{array}\right]$$

By conducting coordinate translation and transformation, the major cutting edge, the minor cutting edge, and the back-side cutting edge are transformed in the same coordinate system [25]. By solving Equations (1), (4), and (7), the actual geometry of the micro-milling tool involved in machining can be obtained as shown in Equation (8).

(8) $$\{\begin{array}{c} x^{2}+y^{2}=\frac{R_{1}{}^{2}+r_{1}{}^{2}+r_{2}{}^{2}}{3}+\frac{u_{1}{}^{2}+u_{2}{}^{2}-2k^{2}}{3}\\ z=k \end{array}$$

The variables *u*_1_, *u*_2_ and *k* can be calculated using the Cramer law, and Equation (8) can also be expressed with Equation (9):

(9) $$\{\begin{array}{c} \left(2-\cos^{2}\gamma -\sin^{2}\delta \right)x^{2}+\left(2-\cos^{2}\delta -\cos^{2}\gamma \right)y^{2}+2\left(\sin\delta \cos\delta +\sin\gamma \cos\gamma \right)xy=R_{1}{}^{2}-r_{1}{}^{2}+r_{2}{}^{2}\\ z=k \end{array}$$

If $\Delta ={\left(\sin\delta \cos\delta +\sin\gamma \cos\gamma \right)}^{2}-\left(2-\cos^{2}\gamma -\sin^{2}\delta \right)\left(2-\cos^{2}\delta -\cos^{2}\gamma \right)>0$, according to the judging criterion of equations, Equation 9 represents a sphere. If Δ <0, Equation (9) represents an ellipsoid, while if Δ = 0, Equation (9) represents a parabola. For different milling tools, according to the parameters of the helix angle and flutes, the corresponding value of Δ can be derived.

For the cutting tool *A*, the helix angle is 15° and the cutting edge angle δ is 45°. Equation (9) can be changed into Equation (10):

(10) $$\{\begin{array}{c} 0.56x^{2}+0.56y^{2}+1.5xy=R_{1}{}^{2}-r_{1}{}^{2}+r_{2}{}^{2}\\ z=k \end{array}$$

Now, Δ= 0.24 > 0, the actually geometry of the cutting tool involved in machining is a sphere with a radius $r=\frac{R_{1}{}^{2}-r_{1}{}^{2}+r_{2}{}^{2}}{0.56}$ in the *X-Y* plane.

For the cutting tool B, the helix angle is 15° and the cutting edge angle δ is 30°, Equation (9) is changed into Equation (11).

(11) $$\{\begin{array}{c} 0.81x^{2}+0.31y^{2}+1.36xy=R_{1}{}^{2}-r_{1}{}^{2}+r_{2}{}^{2}\\ z=k \end{array},$$

where, Δ= 0.21 > 0, the actual geometry was a sphere with a radius $r=\frac{R_{1}{}^{2}-r_{1}{}^{2}+r_{2}{}^{2}}{0.81}$ for the cutting tool *B*.

For the cutting tool *C*, the helix angle is 30°, and the cutting edge angle δ is 30°. Equation (9) is changed into Equation (12).

(12) $$\{\begin{array}{c} x^{2}+0.5y^{2}+1.74xy=R^{2}-r_{1}{}^{2}+r_{2}{}^{2}\\ z=k \end{array}$$

Hence, Δ= 0.26 > 0, the actual geometry was a sphere with a radius $r=\frac{R_{1}{}^{2}-r_{1}{}^{2}+r_{2}{}^{2}}{1}$ for the cutting tool.

For the cutting tool *D*, the helix angle is 30°, and the cutting edge angle δ is 45°. Equation (9) can be changed into Equation (13).

(13) $$\{\begin{array}{c} 0.75x^{2}+0.75y^{2}+1.86xy=R^{2}-r_{1}{}^{2}+r_{2}{}^{2}\\ z=k \end{array}$$

Since Δ= 0.32 > 0, the actual geometry was a sphere with a radius $r=\frac{R_{1}{}^{2}-r_{1}{}^{2}+r_{2}{}^{2}}{0.75}$ for the cutting tool *D*.

From the analyses above, it can be found that the actual geometries of the four cutting tools are all spheres. When all the three cutting edge radii are known, the sphere radius can be determined. When the cutting edge radius remains consistent, the cutting tool *A* has the maximum sphere radius of them all, followed by the cutting tool *D*. The cutting tool *B* and the cutting tool *C* have the minimum sphere radii.

**Figure 6:**
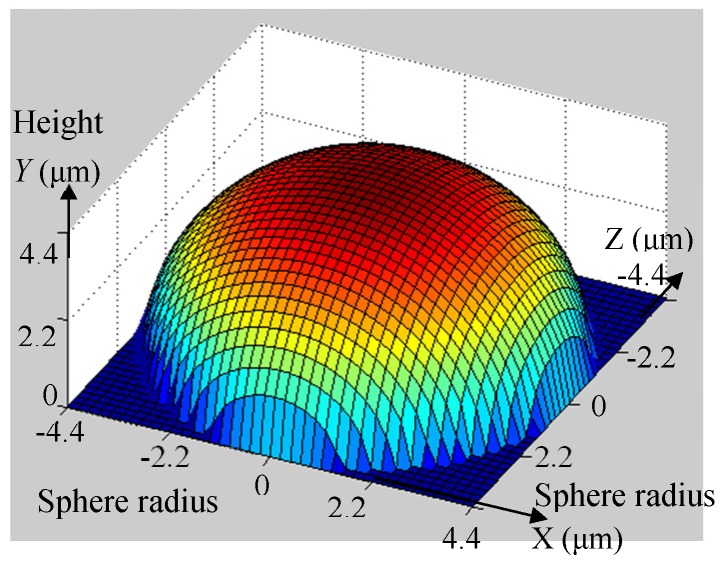
Three-dimensional graphics of actual tool geometries for cutting tool *A*.

Figure 6 shows the actual geometry depicted with the software Matlab 7.0 (The MathWorks, New York, NY, USA) for the cutting tool *A*. According to Equation (10) when the major cutting edge radius is 6 μm, the minor cutting edge radius is 4.9 μm, and the back-side cutting edge radius is 3.8 μm (measured through VHX-600ESO optical microscopy, Keyence, Osaka, Japan).

In Figure 6, it can be seen that the three-dimensional graphics for the cutting tool *A* is a sphere with a radius of 4.4 μm, which means the tip geometry of the actual cutting tool involved in machining is a sphere instead of a very sharp cutting tool with a straight cutting edge involved in machining.

Similar results can be obtained for the other three cutting tools. According to Equations (11)–(13), for the cutting tools *B*, *C*, and *D*, the corresponding sphere radii involved in cutting are 3 μm, 2.45 μm, and 3.3 μm, respectively.

Based on the obtained sphere radii of the four cutting tools, one can find that the sphere radius of the actually geometry increased with the decrease of the helix angle for the same cutting edge radius and flute number. Additionally, the sphere radius of the actually geometry increases with the decreases of the flute number for the same cutting edge radius and flute number.

### 2.2. Surface Roughness Model Considering Swept Mechanisms, Minimum Uncut Chip Thickness, and Cutting Tool Run-Out

**Figure 7:**
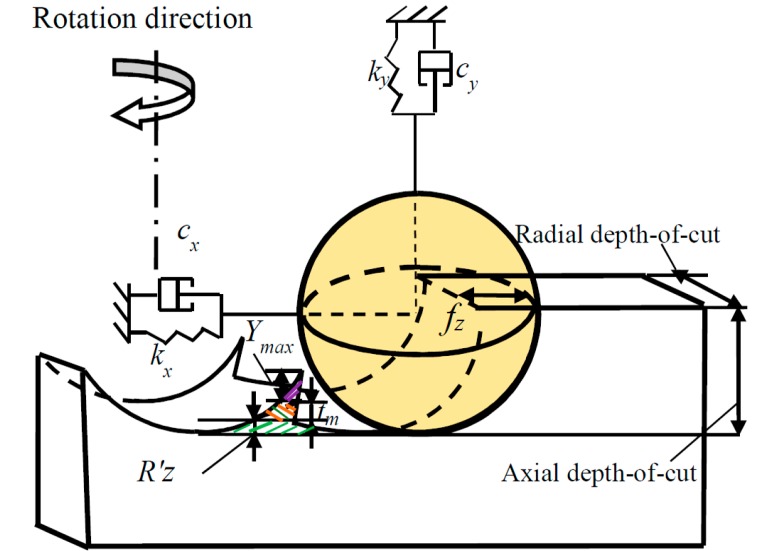
The maximum height of surface roughness deduced in the machining process.

During the machining process, after the workpiece has been decided, there are actually three separate errors that affect surface finish. The first one is cutter quality issues, the second is the tool setup errors, and the third is the machine behavior under load. After machine tools and workpiece have been decided, there are axial and radial run-out errors caused by the cutting tool setup. According to researches of [29,30], one of the significant conditions affecting the machined surface roughness in a stable process is the cutting tool radial run-out. The phenomenon is caused either by tool tilt or displacement with reference to the spindle axis. During the micro milling process, the minimum uncut chip thicknesses also have an influence on the generated surface roughness. In this paper, the prediction model of surface roughness parameters including cutting tool geometry effect, cutting tool run-out, and minimum uncut chip thickness has been built. Figure 7 shows the generated surface considering the three factors mentioned above.

In Figure 7, it can be seen that the maximum height of surface roughness *R_z_* can be formulated as:

(14) $$R_{z}=R_{z}{}^{\prime }+t_{m}+y_{\max}$$

where *R_z_′* is the surface roughness height deduced by the cutting tool geometry, *t_m_* is the minimum uncut chip thickness, *y*_max_ is the cutting tool run-out.

The tool geometry effect on surface roughness height can be stated by the swept mechanism which is explained by the actual volume of the cutting tool involved in the workpiece.

According to Equation (9), the actual geometries of the four different micro-milling tools involved in machining are all part of spheres. Transforming Equation (9) into the *X*-*Y* plane to get the projection form, Equation (15) can be derived.

(15) $$\{\begin{array}{c} \sqrt{2-\cos^{2}\gamma -\sin^{2}\delta }x+\sqrt{2-\cos^{2}\delta -\cos^{2}\gamma }y=\sqrt{R^{2}-r_{1}{}^{2}+r_{2}{}^{2}}\\ z=t \end{array}.$$

It means that the actually geometry is determined by $y=-\frac{\sqrt{2-\cos^{2}\gamma -\sin^{2}\delta }}{\sqrt{R^{2}-r_{1}{}^{2}+r_{2}{}^{2}}}x+\frac{\sqrt{2-\cos^{2}\delta -\cos^{2}\gamma }}{\sqrt{R^{2}-r_{1}{}^{2}+r_{2}{}^{2}}}$ around the *Z*-axis. When the feed rate is *f_z_*, the sweeping volume of the cutting tool involved in the workpiece can be expressed in Equation (16).

(16) $$V={\int_{-r_{1}}^{-f_{z}}\pi \left(-\frac{\sqrt{2-\cos^{2}\gamma -\sin^{2}\delta }}{\sqrt{R_{1}{}^{2}-r_{1}{}^{2}+r_{2}{}^{2}}}x+\frac{\sqrt{2-\cos^{2}\delta -\cos^{2}\gamma }}{\sqrt{R_{1}{}^{2}-r_{1}{}^{2}+r_{2}{}^{2}}}\right)}^{2}dx.$$

When the feed rate is set to be 2 μm per revolution, for the cutting tool *A*, the sweeping volume of the cutting tool involved in machining is 0.02 μm^3^. For the cutting tools *B*, *C*, and *D*, the sweeping volumes involved in machining are 0.0126 μm^3^; 0.012 μm^3^, and 0.016 μm^3^, respectively. So, the cutting tool *A* has the maximum volume involved in cutting, followed successively by the cutting tools *D*, *B*, and *C*. From Equation (15), it also can be seen that, the sweeping volume will increase with the increase of feed rate.

**Figure 8:**
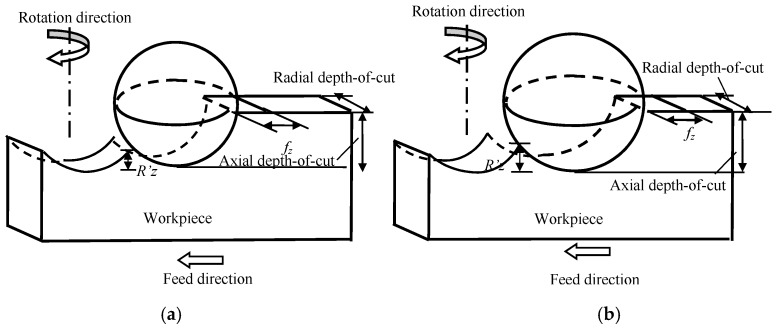
Surface profile generation with different cutting tool geometries. (**a**) Cutting tool with smaller sphere radius; (**b**) cutting tool with larger sphere radius.

Figure 8 illustrates the surface roughness height generated by the sphere cutting tools with different radii. For the cutting tool with a larger sphere radius this leads to a larger sweeping volume according to Equation (16), which means a deeper valley will be generated. The deviation of the profile is used to characterize the surface roughness height.

**Figure 9:**
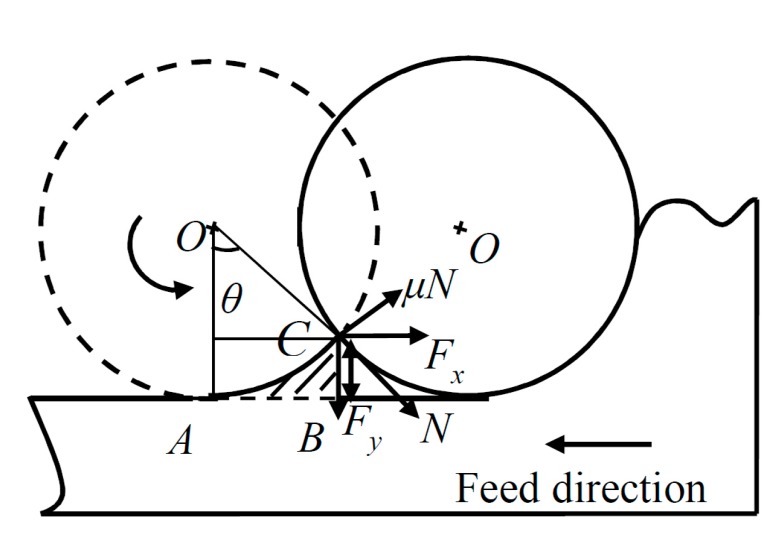
Two-dimensional form of cutting process considering cutting tool geometry.

According to the swept mechanism on the generated surface profile, Figure 8 is converted into a two-dimensional form as shown in Figure 9.

In Figure 9, it can be seen that the first cutting tool path is in the trajectory of *AC*, for the reasons of the geometry of the cutting tool involved in the machining, the shaded part of *ABC* is not removed and the residual height *CB* is treated as surface roughness height *R*’*_z_*.

There are two forces acting at point *C*, horizontal force (i.e., the feed force) *F_x_* and vertical force (i.e., the normal feed force) *F_y_* as shown in Figure 9. The two forces can be divided into normal force *N* and tangential force *μN*, which can be calculated as [8]:

(17) $$N=F_{y}\cos\theta +F_{x}\sin\theta$$

(18) $$\mu N=F_{x}\cos\theta -F_{y}\sin\theta$$

where *μ* is the friction coefficient between the cutting tool and workpiece material.

According to Equations (17) and (18), tan*θ* can be transformed into the following:

(19) $$\tan\theta =\frac{F_{x}-\mu F_{y}}{\mu F_{x}+F_{y}}.$$

*R*’*_z_* can be calculated as shown in Equation (20)

(20) $$Rz^{\prime }=R\left(1-\cos\theta \right)=R\left(1-\frac{1}{\sqrt{1+\tan^{2}\theta }}\right).$$

Substituting Equation (19) into Equation (20) gives:

(21) $$Rz^{\prime }=R\left(1-\frac{\mu F_{x}+F_{y}}{\sqrt{\left(1+\mu^{2}\right)\left(F_{x}{}^{2}+F_{y}{}^{2}\right)}}\right).$$

In Equation (21), *R*’*_z_* can be calculated if the radius of the sphere, the ratio of *F_y_*/*F_x_* and the friction coefficient *μ* are known.

**Figure 10:**
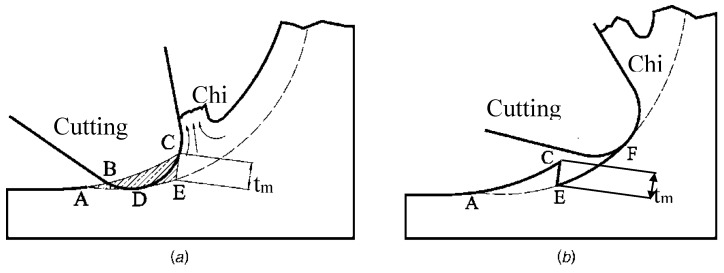
Effect of minimum uncut chip thickness on surface generation when the cutting tool first cuts into the workpiece (**a**) and further (**b**).

The minimum uncut chip thickness in the micro milling process can lead to the change of surface height as shown in Figure 10.

In Figure 10a, when the cutting tool first cuts into the workpiece, the trajectory of the cutting tool is along the circular path ABC. When the cutting tool cuts into the workpiece again, the actual feed is zero at point A, and then begins to increase. The trajectory of the cutting tool is along the circular path ADE. After that there is chip formation. During this cutting process, the workpiece materials in the area of ABCDE will not format chip and only undergo a ploughing effect. With the cutting tool and further cuts in the workpiece, as shown in Figure 10b, the ploughed materials accumulate in ACE. The generated surface begins from point E.

According to Son [9], the minimum uncut chip thickness can be expressed in Equation (22):

(22) $$t_{m}=R\left(1-\cos\left(\frac{\pi }{4}-\frac{\beta }{2}\right)\right),$$

where *β* is the friction angle between cutting tool and workpiece.

**Figure 11:**
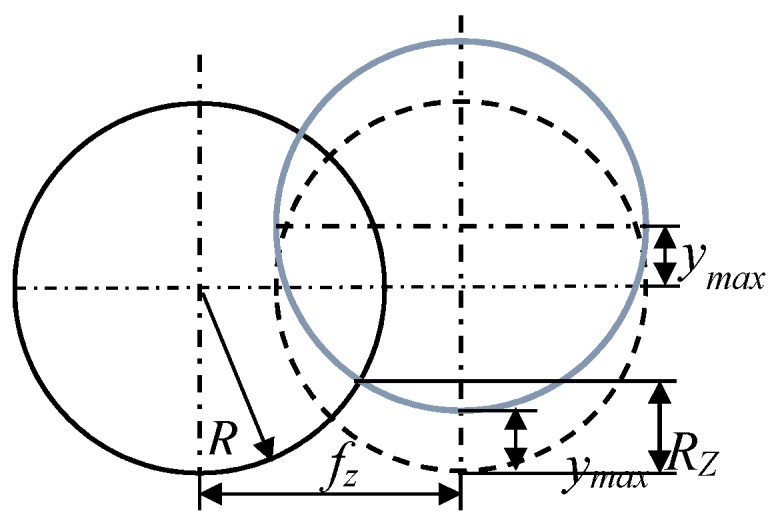
Determination of cutting tool run-out during the cutting process.

The cutting tool dynamic run-out effect on surface roughness height is shown in Figure 11.

In order to calculate the dynamic run-out of the cutting tool, it is necessary to solve the differential motion equation:

(23) $$m\cdot \ddot{y}\left(t\right)+c\cdot \dot{y}\left(t\right)+k\cdot y\left(t\right)=F_{i}\left(t\right)$$

where *m*, *c*, and *k* are modal mass, damping, and stiffness matrixes, respectively. The *F_i_*(*t*) is the resulting vector of the cutting force components, and *y* is the dynamic run-out.

**Figure 12:**
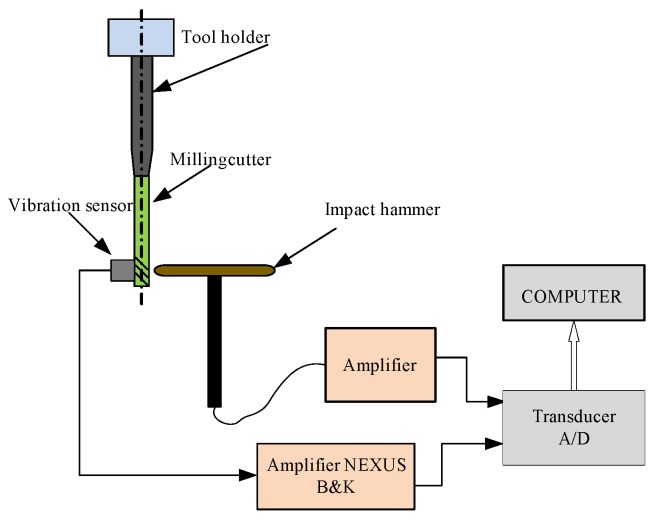
The schematic diagram of the impact test configuration.

The left side of Equation (23) is determined using an impact test. Figure 12 shows the scheme of impact test configuration. The determined values of modal parameters are valid only for a particular machine-collet-tool system.

Considering force variation of each tooth, a newΔ*e_i_* part which considers the cutting tool radial run-out is introduced.

In the *F_f_* feed and *F_fN_* normal feed direction, the following Equations (24) and (25) are valid.

(24) $$\Delta e_{f}=\left(\frac{k_{dFf}-1}{2k_{dFf}}\right)\cdot \sin\left(\frac{\pi \cdot n\cdot z}{30}+\frac{\pi }{2}\right)+\left(\frac{k_{dFf}+1}{2k_{dFf}}\right)$$

(25) $$\Delta e_{fN}=\left(\frac{k_{dFfN}-1}{2k_{dFfN}}\right)\cdot \sin\left(\frac{\pi \cdot n\cdot z}{30}+\frac{\pi }{2}\right)+\left(\frac{k_{dFfN}+1}{2k_{dFfN}}\right)$$

where *k_dfN_* and *k_dFfN_* are dynamic coefficients empirically determined, *n* is the spindle speed and *z* is the number of cutting teeth.

Finally, equations that define instantaneous values of feed and normal feed force components including radial run-out of the milling process, can be derived:

(26) $$F_{f\Delta e}=\Delta e_{f}\cdot F_{f}$$

(27) $$F_{fN\Delta e}=\Delta e_{fN}\cdot F_{fN}$$

The ultimate form of Equation (23) can be written as:

(28) $$m\cdot \ddot{y}\left(t\right)+c\cdot \dot{y}\left(t\right)+k\cdot y\left(t\right)=F_{i\Delta e}\left(t\right)$$

The determined values of the dynamic force were submitted into Equation (28), which is solved using a 4-th order Runge-Kutta algorithm. The cutting tool run-out in the feed direction was neglected because of its marginal influence on the generation of surface roughness parameters. After calculation, the cutting tool run-outs in the direction perpendicular to the machined surface are obtained.

## 3. Experiments

**Figure 13:**
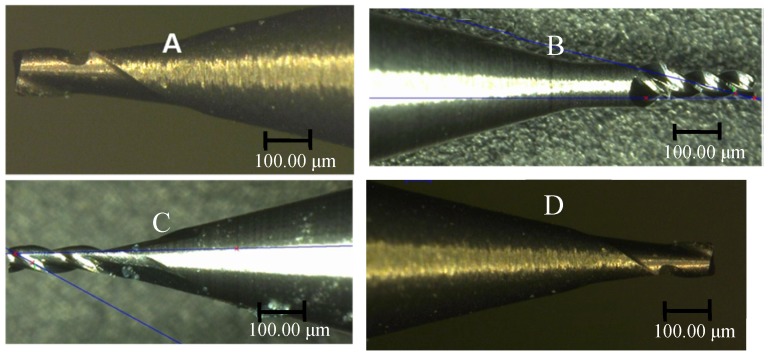
The applied four different cutting tools. (**a**) two flutes with a helix angle of 15°; (**b**) three flutes with a helix angle of 15°; (**c**) three flutes with a helix angle of 30°; (**d**) two flutes with a helix angle of 30°.

**Figure 14:**
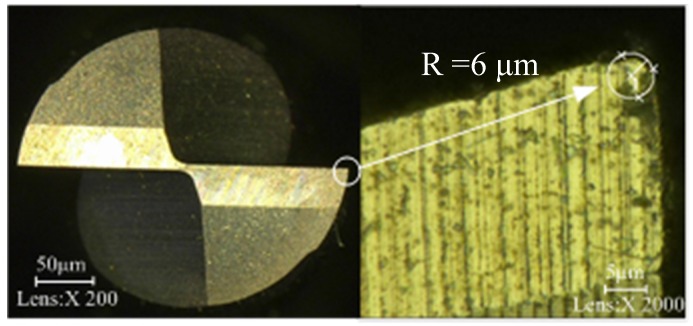
Measuring process of the cutting edge radius.

Four different micro-milling tools with different shapes shown in Figure 13 were used for the experiments. As shown in Figure 13, all the four cutting tools used in experiments are made of ultra-fine grain cemented carbide and used for end milling with cutting tool flute diameter 0.35 mm (made by KANELER Company of Germany, Munchen, Germany). The cutting tool *A* has two flutes with a helix angle of 15°. The cutting tool *B* has three flutes with a helix angle of 15°. The cutting tool *C* has three flutes with a helix angle of 30°. The cutting tool *D* has two flutes with a helix angle of 30°. All the helix lengths for the four cutting tools are 2 mm and the minor cutting edge radius was tested to be 6 μm. Figure 14 shows the measuring process of the cutting edge radius.

The details of the cutting tool used for experiments are shown in Table 1.

**Figure 15:**
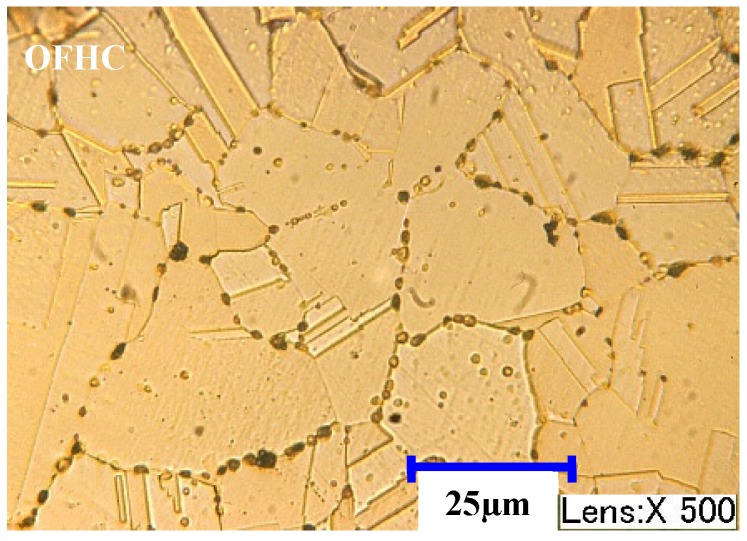
Micro structure for Oxygen Free High Conductivity Copper (OFHC).

Experiments were conducted at the KERN 2522 milling center. The workpiece material used in this study is OFHC, which is typically used for structural and thermal applications such as welding/brazing rods and heat exchanger tubing. The micro structure of the workpiece is shown in Figure 15. The average grain size of the material was tested to be 50 μm using the linear intercept method, which can be used to guide the selection of machining parameters.

In the research carried out, surface roughness parameters were measured for two different feed rates: 2 μm per revolution and 3 μm per revolution. The following cutting parameters were employed in dry machining tests: cutting speed 275 mm/s, the axial depth-of-cut is 100 μm and the radial depth-of-cuts are 75 μm and 80 μm. At this size scale, the tool-workpiece interaction occurred entirely within either a single crystal or a few crystals.

**Figure 16:**
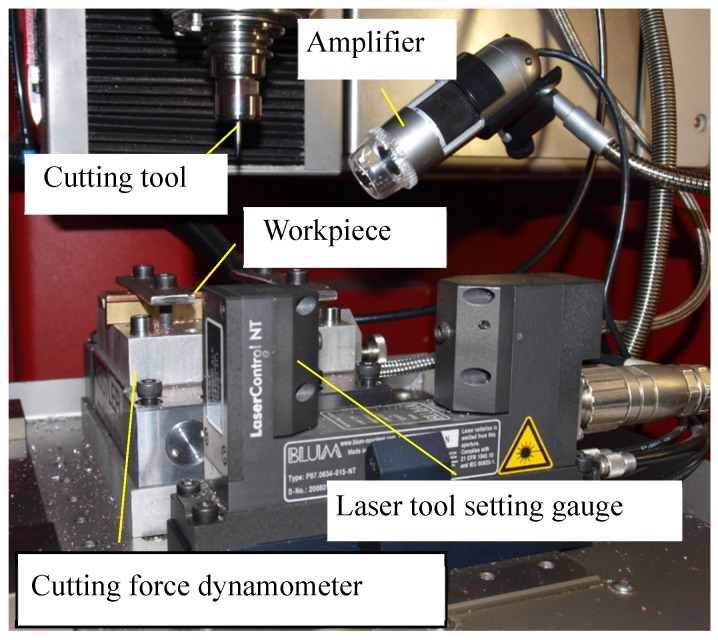
Micro milling center for cutting experiments.

The surface roughness was determined using a Veeco WykoNT9300 white light interferometer (VEECO, Los Angels, CA, USA). During the cutting process, the effect of the cutting tool geometry on the cutting force was also investigated. The cutting force was measured with a Kistler 9129AA dynamometer (Kistler, Bern, Switzerland) as shown in Figure 16.

**Figure 17:**
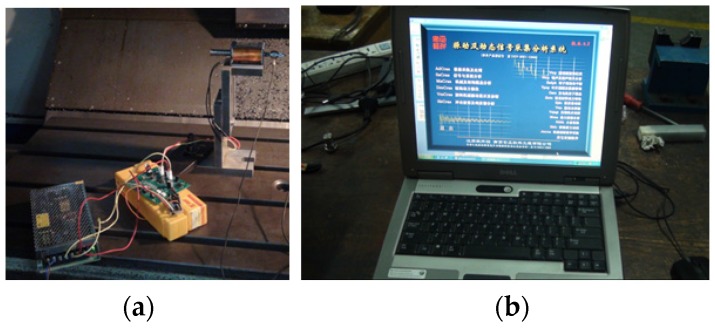
Impact test configuration. (**a**) Impact hammer for modal test; (**b**) signal acquisition and analysis system.

The impact tests were conducted to identify the dynamic parameters of the cuttings system. Figure 17a shows the impact hammer used for the modal test and Figure 17b shows the vibration and dynamic signal acquisition and analysis system. Orthogonal cutting experiments were also conducted to test the friction coefficient between OFHC and cemented carbide.

## 4. Experimental Results and Discussions

### 4.1. Cutting Force and Friction Coefficient

**Figure 18:**
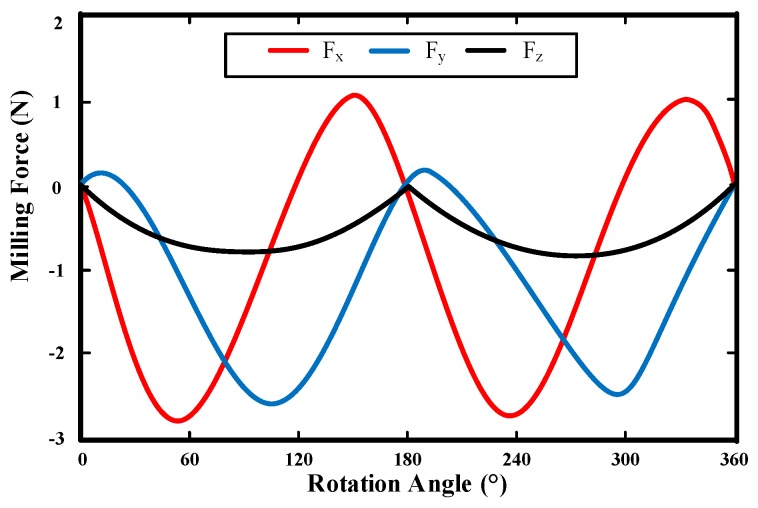
Instantaneous cutting force for cutting tool *A*.

Figure 18 shows the instantaneous cutting forces for cutting tool *A* in the *X*, *Y*, and *Z* directions. *F_x_* is the cutting force in the feed direction, *F_y_* is the cutting force in the normal feed direction and *F_z_* is the cutting force in the axial direction.

Table 2 shows the instantaneous maximum cutting forces for the four different cutting tools. It can be seen that the cutting force is relatively small compared to that in the macro cutting process. The cutting force in the feed direction is always the largest, followed by the cutting force in the normal feed direction and the cutting force in the axial direction. From Table 2, it also can be seen that cutting tool *C* corresponds to the minimum cutting force, followed by cutting tool *B*, and cutting tool *D*; the cutting tool *A* corresponds to the largest cutting force.

**Figure 19:**
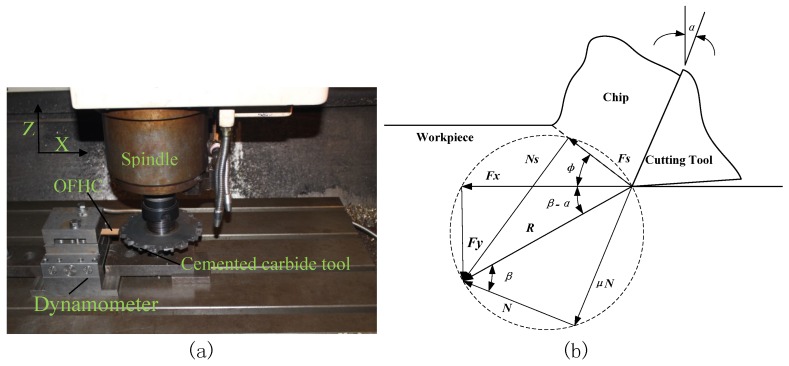
Friction test for cutting tool and workpiece. (**a**) Physical map; (**b**) model map.

Figure 19 shows the friction test process. Orthogonal cutting experiments were conducted to make the rake angle *α* equal 0°. The workpiece is OFHC and the material of the cutting tool is cemented carbide which is the same for the four different cutting tools.

As shown in Figure 19, *μ* = tan*β* where *β* is the friction angle. The friction coefficient can be calculated as follows:

(29) $$\mu =\frac{\mu N}{N}=\frac{F_{x}\sin\alpha +F_{y}\cos\alpha }{F_{x}\cos\alpha -Fy\sin\alpha }=\frac{F_{y}+F_{x}\tan\alpha }{F_{x}-F_{y}\tan\alpha }$$

In the orthogonal cutting process, *α* is 0°. The friction coefficient between OFHC and cemented carbide was tested to be 0.29.

According to the experimental results, the ratio of *F_y_*/*F_x_* for cutting tools *A*, *B*, *C*, and *D* are 0.98, 0.97, 0.97, and 0.96. Therefore, the surface roughness is calculated to be 890 nm, 680 nm, 620 nm, 796 nm respectively.

The minimum uncut chip thickness for the four different cutting tools are determined to be 0.88, 0.6, 0.49, and 0.66 μm respectively for cutting tool *A*, *B*, *C*, and *D* based on Equation (22).

### 4.2. Modal Parameters and Cutting Tool Run-Out

On the basis of known input signal parameters (identified by impact hammer and force sensor), the frequency response function is obtained, and thus the modal parameters.

For cutting tool *A*: *m_a_* = 0.027 Ns^2^/m; *c_a_* = 69 Ns/m; *k_x_* = 9,724,639 N/m. For cutting tool *B*: *m_a_* = 0.018 Ns^2^/m; *c_a_* = 54 Ns/m; *k_x_* = 8,796,642 N/m. For cutting tool *C*: *m_a_* = 0.017 Ns^2^/m; *c_a_* = 47 Ns/m; *k_x_* = 8,164,487 N/m. For cutting tool *D*: *m_a_* = 0.023 Ns^2^/m; *c_a_* = 61 Ns/m; *k_x_* = 9,185,764 N/m.

**Figure 20:**
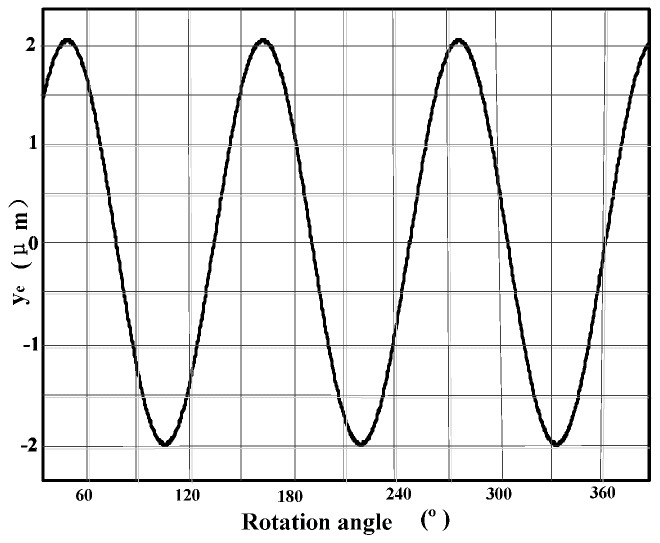
Time course of cutting tool run-out for cutting tool *A*.

Figure 20 shows the time course of the envelope of the cutter run-out, calculated in MatLab software based on Equation (28) for cutting tool *A*.

From Figure 20, it can be seen that the maximum height of the envelope calculated in MatLab software for cutting tool *A* is equal to *y_emax_* = 4 μm. The run-outs are equal to 3.7, 3.6, and 3.9 μm respectively for cutting tool *B*, *C*, and *D*.

The surface roughness height *R_z_* can be calculated according to the results mentioned above. The average surface roughness *R_a_* is developed on the assumption that it is equal to the mean arithmetic deviation of the profile line from the average line. In order to determine the average line of the surface irregularity profile, the center of gravity of the area below the profile was calculated. The Derive 6 software was applied and the average surface roughness *R_a_* can be calculated as follows:

(30) $$R_{a}=\frac{3\cdot R_{z}\left(3\cdot R_{z}+4R\right)\left(3\cdot R_{z}{}^{2}+4R\cdot R_{z}-20R^{2}\right)}{1000R^{3}}$$

### 4.3. Surface Roughness Analysis

**Figure 21:**
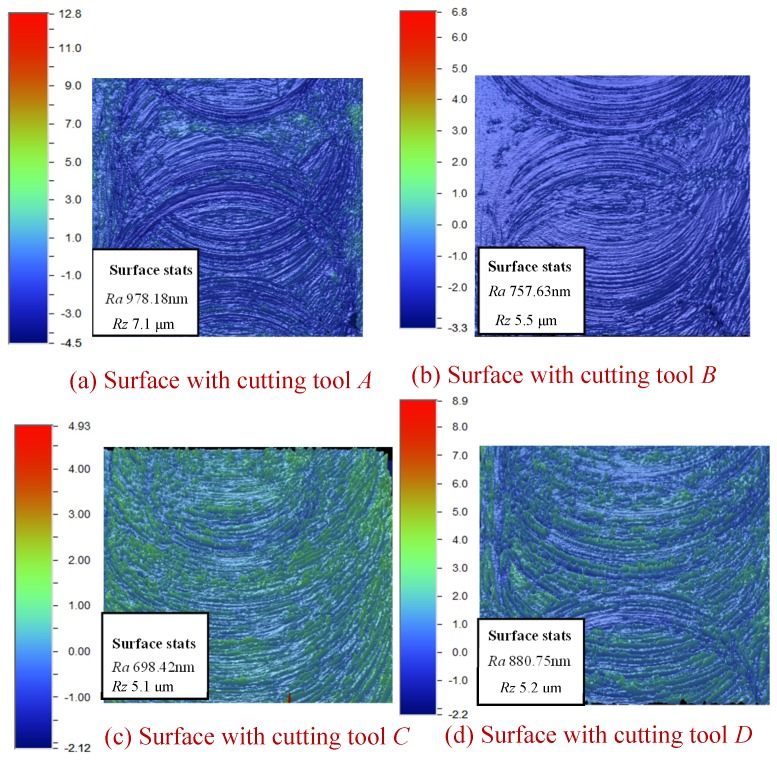
Machined surface with four different cutting tools.

The 3D images of the surface topography are shown in Figure 21.

As seen in Figure 21, the surface roughness is tested by rectangular truncating of the surface center to make sure the partial edge of the machined surface is not involved in the measurement of surface roughness. The different colors in Figure 21 show different heights of the surface. It can be seen in Figure 21 that the smallest surface roughness parameters correspond to the cutting tool *C* with *R_a_* 698.42 nm, *R_z_* 5.1 μm followed by the cutting tool *B* with *R_a_* 757.63 nm, *R_z_* 5.5 μm**, the cutting tool *D* with *R_a_* 880.75 nm, *R_z_* 5.2 μm. The worst surface roughness corresponds to the cutting tool *A* with *R_a_* 978.18 nm, *R_z_* 7.1 μm.

From Figure 21, it can be seen that the machined surface textures are uneven when using cutting tool *A* and cutting tool *D*. The machined surfaces on both sides are coarse distribution, and the intermediate section is dense distribution. The consistency between the two feeds per tooth in micro per revolution is poor for the two cutting tools. While by using the cutting tool *B* and the cutting tool *C*, the machined surface textures are relatively uniform. The surfaces generated between the two feeds per tooth in micro per revolution maintain consistency.

**Figure 22:**
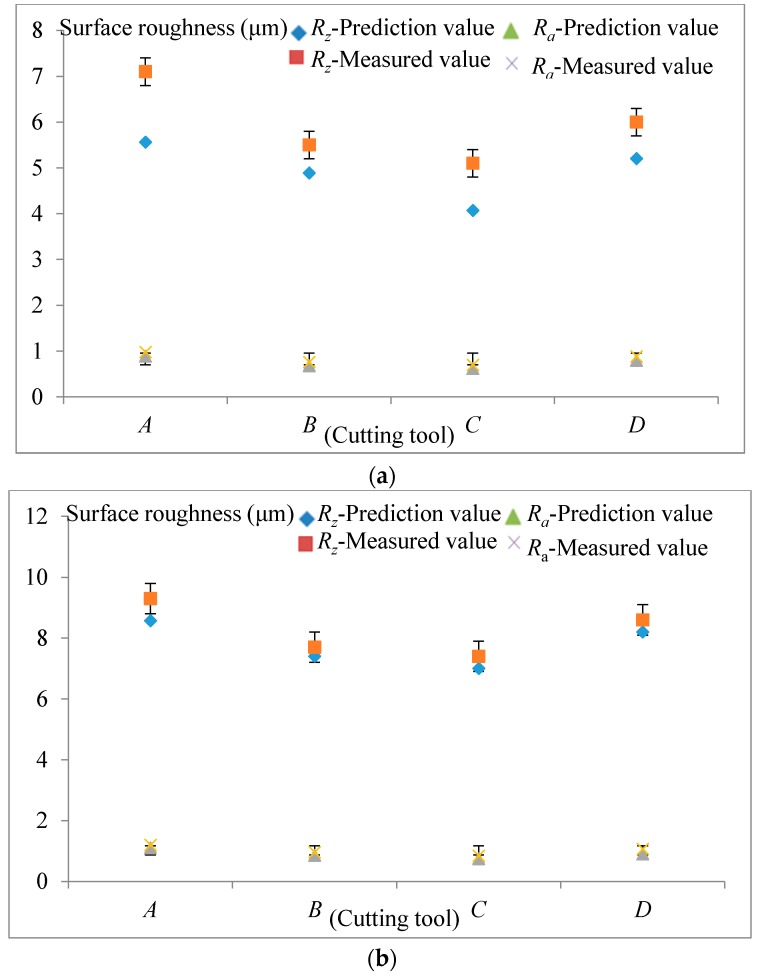

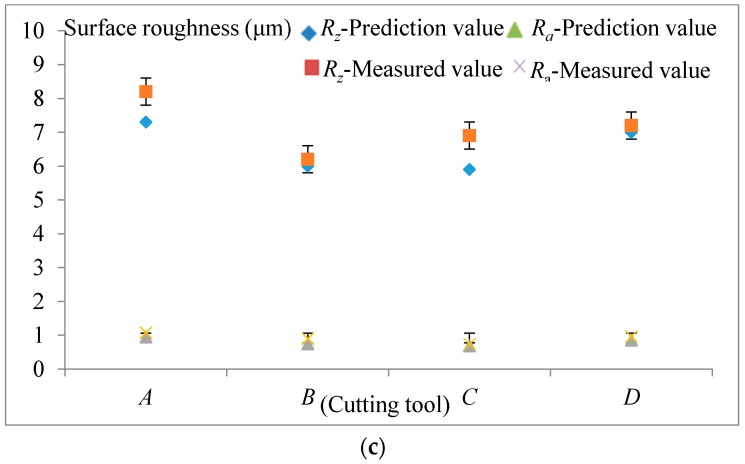
Surface roughness parameters for different cutting tools. (**a**) Values of *R_z_* and *R_a_* under feed rate 2 μm per revolution, radial depth-of-cut 75 μm; (**b**) Values of *R_z_* and *R_a_* under feed rate 3 μm per revolution, radial depth-of-cut 75 μm; (**c**) Values of *R_z_* and *R_a_* under feed rate 2 μm per revolution, radial depth-of-cut 80 μm.

The measured surface roughness parameters *R_z_* and *R_a_*, and the calculated (using Equations (14) and (23)) surface roughness parameters are depicted in Figure 22.

As can be observed in Figure 22, the measured surface roughness parameters *R_a_* and *R_z_* which are confirmed by considering values of error bars are higher than the calculated results for the four different cutting tools. It can be seen that the values of *R_z_* are 7–8 times higher from *R_a_*. The calculated and the measured surface roughness heights *R_z_* are close to the cutting tool run-outs. This means that during the micro-milling process, the height of the generated surface is mainly affected by the cutting tools run-out. This results from geometrical errors of the machine-tool system. The measured average surface roughness *R_a_* is close to the calculated values, which is also close to the calculated surface roughness values only considering the swept mechanism effect of the cutting tool.

Figure 22 also shows the influence of feed rate and radial depth-of-cut on the surface roughness parameters. It can be seen that surface becomes rough when the feed rate increases due to the increase of the overlap between cutting paths. It can also be induced that the increase of radial depth-of-cut also results in more surface roughness values. The sensitivity of surface roughness values to process parameters shows that the feed rate has more significance than radial depth-of-cut. From Figure 22, it also can be seen that for the average surface roughness value there exists a certain variation to be considered. It is generally in the range of 0.7–1.2 μm.

From Figure 22, it can be seen that the surface roughness parameters *R_z_* and *R_a_* will be greater if the sphere radius is larger under the same cutting parameters including depth-of-cut and feed rate, which agrees with the results formed by Yuan [8] and Liu [31]. In their research, they pointed out that the sharper the cutting edge, the smaller the surface roughness. This is the same as our conclusion. The difference is that the actual geometry of the cutting tool involved in machining is a sphere according to our calculation, while for Yuan [8] and Liu [31], only the cutting edge radius was considering to be involved in the machining. Thus, the increase of the volume of the cutting tool involved in the workpiece, which can be determined by the cutting edge radius and cutting tool flutes, would lead to an increase of the machined surface roughness. The more volume of the cutting tool involved in the workpiece, the coarser is the surface roughness produced. The same conclusions can be obtained by changing feed rates or radial depth-of-cut as shown in Figure 22.

**Figure 23:**
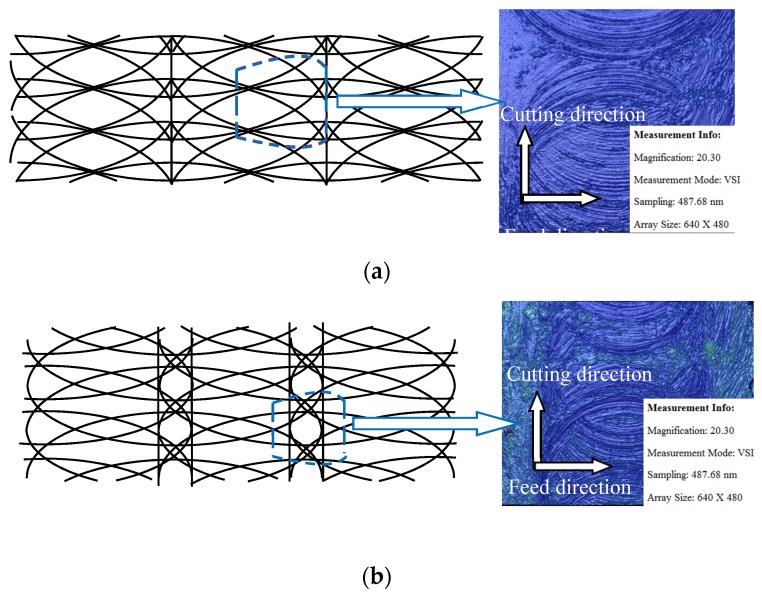
Comparison between simulated machined surface and experimental measurement results. (**a**) Surface topography under smaller sphere radius (cutting tool *B*); (**b**) Surface topography under larger sphere radius (cutting tool *A*).

Figure 23 shows the simulated results of the machined surface topography using two spheres with different radii, under the same feed rate and depth-of-cut.

The simulated pattern on the left side based on the process parameters and sphere radii can characterize the basic features of the measured ones. It should be pointed out that the simulated surface pattern is only based on tool path kinematics, while the surface material deformation is not incorporated. Therefore, a difference between the simulated pattern and the experimental ones is expected. The left part of Figure 23 shows the simulated machined surface using two spheres with radii of 3 and 4.4 μm constructed with the same frequency which means under the same feed rate and depth-of-cut. It is found that a more uniform texture distribution of the machined surface bottom can be obtained with a smaller sphere radius. While for the cutting tool with large radius, the texture of the machined surface bottom was in the same horizontal position. The texture of the middle part was distributed evenly, and the marginal part of the texture had obvious variations. The simulated pattern shows the basic characteristics of the milling surface topography. This is in good agreement with the experimental results. The above analysis of the surface topography for the four different cutting tools suggested that the geometric effect of the cutting tool and process conditions affect the surface roughness together. With the same process conditions, more cutting tool flutes or a larger helix angle are preferred to obtain machined surfaces with uniform texture distribution, which correspond to a smaller sphere radius of the actual tool geometry.

## 5. Conclusions

In this paper, four different micro-end-milling tools were examined to determine the influence of the mechanism of cutting tool geometry on surface roughness by both experimental and theoretical methods. A set of cutting experiments using these four different cutting tools were conducted and the surface topography was obtained. The following conclusions can be drawn:

(1) The established sweeping model shows that the cutting edge radius, helix angle, and the number of cutting tool flutes can be used to decide the actual geometry. When the critical value determined by the cutting tool parameters is positive, the geometry involved in machining will be a sphere, conversely, but the geometry will be an ellipsoid when negative. The results were valid throughout the experimental results. The outcome of this research should bring about new methodologies on improving surface roughness and preparing cutting tools.
(2) Both the cutting tool geometries and cutting process conditions have effects on the machined surface roughness. More cutting tool flutes or a larger helix angle which involve less volume of the cutting tool in the workpiece are preferred to obtain machined surfaces with uniform texture distribution.
(3) The effect of cutting tool run-out and minimum uncut chip thickness was considered on surface roughness parameters. Results show that during the process of micro-milling, the height of the generated surface *R_z_* is mainly affected by the cutting tools run-out. The average surface roughness *R_a_* is close to the surface roughness values only when considering the swept mechanism effect of the cutting tool.

## Figures and Tables

**Table 1:** Details of cutting tool used for experiments.

Cutting Tool	Flute Diameters	Helix Length	Helix Angle	Tool Fluted	Materials
*A*	0.35 mm	2 mm	15°	2	cemented carbide
*B*	0.35 mm	2 mm	15°	3	cemented carbide
*C*	0.35 mm	2 mm	30°	3	cemented carbide
*D*	0.35 mm	2 mm	30°	2	cemented carbide

**Table 2:** The instantaneous cutting forces for different cutting tools.

Cutting Tool	*F_x_* (N)	*F_y_* (N)	*F_z_* (N)
*A*	2.93	2.89	0.72
*B*	2.83	2.76	0.62
*C*	2.81	2.70	0.41
*D*	2.90	2.78	0.67
